# Spatial–temporal variability and health impact of particulate matter during a 2019–2020 biomass burning event in Southeast Asia

**DOI:** 10.1038/s41598-022-11409-z

**Published:** 2022-05-10

**Authors:** Murnira Othman, Mohd Talib Latif, Haris Hafizal Abd Hamid, Royston Uning, Thipsukon Khumsaeng, Worradorn Phairuang, Zawawi Daud, Juferi Idris, Nurzawani Md Sofwan, Shih-Chun Candice Lung

**Affiliations:** 1 Institute for Environment and Development (LESTARI), Universiti Kebangsaan Malaysia, 43600 Bangi, Selangor Malaysia; 2 Centre for Toxicology and Health Risk Studies, Faculty of Health Sciences, Universiti Kebangsaan Malaysia, Jalan Raja Muda Abdul Aziz, 50300 Kuala Lumpur, Malaysia; 3 Department of Earth Sciences and Environment, Faculty of Science and Technology, Universiti Kebangsaan Malaysia, 43600 Bangi, Selangor Malaysia; 4 Institute of Oceanography and Environment (INOS), Universiti Malaysia Terengganu, 21030 Kuala Nerus, Terengganu Malaysia; 5 Department of Physics and Materials Science, Faculty of Science, Chiang Mai University, 239, Huay Kaew Road, Muang District, Chiang Mai, 50200 Thailand; 6 Faculty of Geosciences and Civil Engineering, Institute of Science and Engineering, Kanazawa University, Kanazawa, Ishikawa, 920-1192 Japan; 7 Faculty of Civil Engineering and Built Environment, Universiti Tun Hussein Onn Malaysia (UTHM), Parit Raja, 86400 Batu Pahat, Johor Malaysia; 8 Faculty of Chemical Engineering, College of Engineering, Universiti Teknologi MARA (UiTM), Sarawak Branch, Samarahan Campus, 94300 Kota Samarahan, Sarawak Malaysia; 9 Faculty of Chemical Engineering, College of Engineering, Universiti Teknologi MARA (UiTM), Selangor Branch, 40450 Shah Alam, Selangor Malaysia; 10 Department of Environmental Health, Faculty of Health Sciences, Universiti Teknologi MARA (UiTM), Sarawak Branch, Samarahan Campus, 94300 Kota Samarahan, Sarawak Malaysia; 11 Research Center for Environmental Changes, Academia Sinica, Taipei, Taiwan, ROC

**Keywords:** Environmental chemistry, Environmental impact, Atmospheric chemistry

## Abstract

To understand the characteristics of particulate matter (PM) in the Southeast Asia region, the spatial–temporal concentrations of PM_10_, PM_2.5_ and PM_1_ in Malaysia (Putrajaya, Bukit Fraser and Kota Samarahan) and Thailand (Chiang Mai) were determined using the AS-LUNG V.2 Outdoor sensor. The period of measurement was over a year from 2019 to 2020. The highest concentrations of all sizes of PM in Putrajaya, Bukit Fraser and Kota Samarahan were observed in September 2019 while the highest PM_10_, PM_2.5_ and PM_1_ concentrations in Chiang Mai were observed between March and early April 2020 with 24 h average concentrations during haze days in ranges 83.7–216 µg m^−3^, 78.3–209 µg m^−3^ and 57.2–140 µg m^−3^, respectively. The average PM_2.5_/PM_10_ ratio during haze days was 0.93 ± 0.05, which was higher than the average for normal days (0.89 ± 0.13) for all sites, indicating higher PM_2.5_ concentrations during haze days compared to normal days. An analysis of particle deposition in the human respiratory tract showed a higher total deposition fraction value during haze days than on non-haze days. The result from this study indicated that Malaysia and Thailand are highly affected by biomass burning activity during the dry seasons and the Southwest monsoon.

## Introduction

Southeast Asia, located in the tropics, is a region with a high proportion of developing countries. Some areas in Southeast Asian countries, especially in Sumatra, Kalimantan and northern Thailand, have been cleared for large scale commercial agriculture and medium to small scale farming. Large amounts of residue from agricultural-based economic activities, such as rice straw, maize, sugarcane, and other crops are burned in Thailand and other Southeast Asian countries^1^ producing high levels of air pollutants in the form of aerosols and particulate matter (PM)^2,3^. Specifically, both agricultural waste burning and uncontrolled biomass burning with the additional factor of atmospheric inversion in the mountainous areas of northern Thailand are the main causes of severe air pollution in this location^4,5^. In southern Southeast Asia, particularly Malaysia and Indonesia, biomass burning activity is associated with peatland fires. Fires in peatland are a serious threat to air quality and occur during the dry season and the Southwest monsoon^6,7^.

Air quality deteriorates as a result of high levels of air pollution caused by climate change, increased biogenic emissions, and changes in meteorological parameters, all of which also contribute to haze episodes^8^. Severe haze episodes were also suggested to be associated with extreme weather conditions, i.e. droughts induced by El Niño-Southern Oscillation (ENSO)^9,10^. Haze episodes are associated with low visibility (< 10 km) due to high atmospheric loading of solid particulates whereas low visibility during haze episode has been suggested to be as low as 1.0–5.8 km and can be further reduced for the areas near the biomass burning location^11–13^. According to a study by Xing et al.^14^ hazy conditions caused by elevated pollutant concentrations during biomass burning events in Southeast Asia were not only impacted locally but also transboundary, which was a significant source of increasing PM_2.5_ concentrations in southern China. Previous studies also revealed the impact of haze pollution on the environment related to microbial community change and airborne fungal abundance^15,16^, an increase in caterpillar mortality and interference in butterfly development^17^ as well as reduction of total rice and wheat production^18^. It is undeniable that haze episodes have a significant impact on the environment, ecology and animal habitats and human health.

The human health impacts of haze episodes are usually related to the abundance of particulate matter (PM) suspended in the air. Smaller size PM is associated with a high potential risk of adverse health effects as it has higher surface area to volume ratios compared to larger size particles and small particles can penetrate deep into the alveoli and extra-pulmonary tissue^19,20^. Exposure to PM, particularly particles with diameters of less than 2.5 µm (PM_2.5_), during haze episodes has been determined to be a major cause of chronic obstructive pulmonary disease (COPD)^21,22^, increased mortality risk^23^, cardiovascular morbidity and mortality including cardiac death^24^, lung cancer and reduced lung function^25,26^, diabetes mellitus^27^ and premature death^28^.

Southeast Asian countries face severe air pollution events and haze episodes during the dry season, making it critical to understand the characteristics of PM, particularly that associated with biomass burning emissions^2^. An investigation of PM levels in northern Thailand and Malaysia during the entire season and monsoon could provide valuable insight into how both areas have been exposed to different types of biomass burning sources. This study aims to evaluate the spatial–temporal characteristics of three different sizes of PM (PM_10_, PM_2.5_ and PM_1_) in four different measurement sites in Malaysia and Thailand, Southeast Asia. The characteristics of the PM, which vary with biomass burning activities and seasonal variation, at each monitoring sites are evaluated. Deposition of particles in human respiratory tract was also determined for haze and non-haze scenarios. The results from this study will provide understanding for pollution control strategies, especially during the dry season in Southeast Asia, and can help to reduce the exposure of the population to PM.

## Results

### Spatial–temporal characteristics of PM_10_, PM_2.5_ and PM_1_

**Figure 1:**
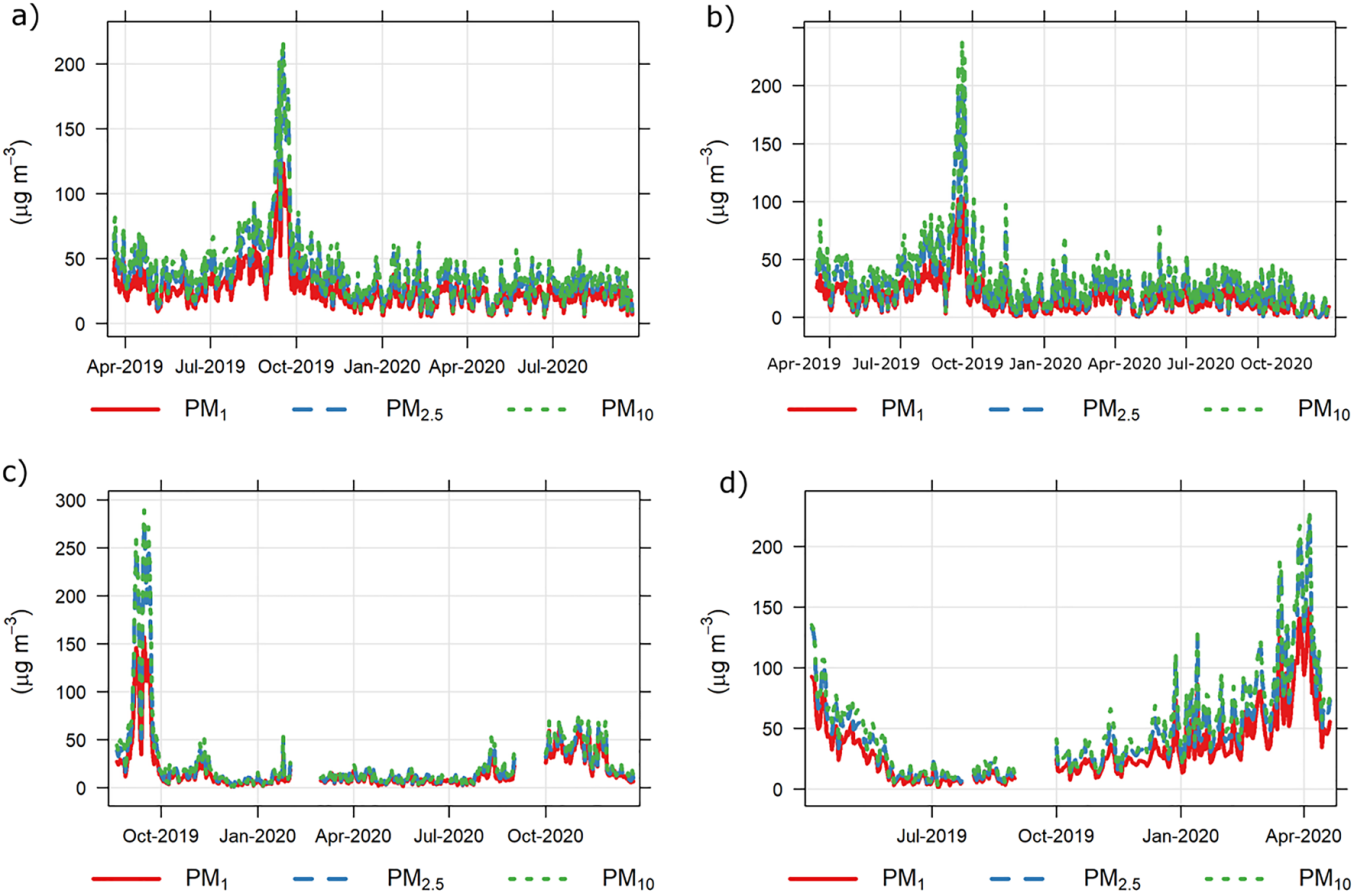
Daily average concentration of PM_10_, PM_2.5_ and PM_1_ in (**a**) Putrajaya, (**b**) Bukit Fraser, (**c**) Kota Samarahan and (**d**) Chiang Mai.

The 24 h daily average trends of PM_10_, PM_2.5_ and PM_1_ during the measurement campaign in the year 2019–2020 are shown in Fig. 1 and a descriptive summary is given in Table S1. Overall, the ranges of PM_10_ concentrations for the whole measurement period were 7.08–217 µg m^−3^ (Putrajaya), 1.11–238 µg m^−3^ (Bukit Fraser), 1.19–289 µg m^−3^ (Kota Samarahan), 3.24–216 µg m^−3^ (Chiang Mai), for PM_2.5_ were 6.88–208 µg m^−3^ (Putrajaya), 0.62–203 µg m^−3^ (Bukit Fraser), 0.99–278 µg m^−3^ (Kota Samarahan) and 2.74–209 µg m^−3^ (Chiang Mai) and for PM_1_ were 5.74–123 µg m^−3^ (Putrajaya), 0.33–102 µg m^−3^ (Bukit Fraser), 0.73–156 µg m^−3^ (Kota Samarahan) and 1.72–140 µg m^−3^ (Chiang Mai). PM_10_, PM_2.5_ and PM_1_ had similar trends at all measurement sites, with peak concentrations of PM_10_, PM_2.5_ and PM_1_ observed in the middle of September 2019 for Putrajaya, Bukit Fraser and Kota Samarahan and two peaks in April 2019 and 2020 in Chiang Mai. All of the peak concentrations of PM_10_, PM_2.5_ and PM_1_ were observed to exceed 100 µg m^−3^ at all monitoring sites where the concentrations of PM_10_, PM_2.5_ and PM_1_ were the highest at Kota Samarahan with a maximum average 24 h concentration of 289 µg m^−3^, 278 µg m^−3^ and 156 µg m^−3^, respectively. A stable increase in PM concentrations from the beginning of 2020 to March 2020 with an increase of 1.2 times the monthly concentration was determined for Chiang Mai, no further increase was observed in April 2020 and a decrease in concentration was observed in the second week of April 2020.

**Figure 2:**
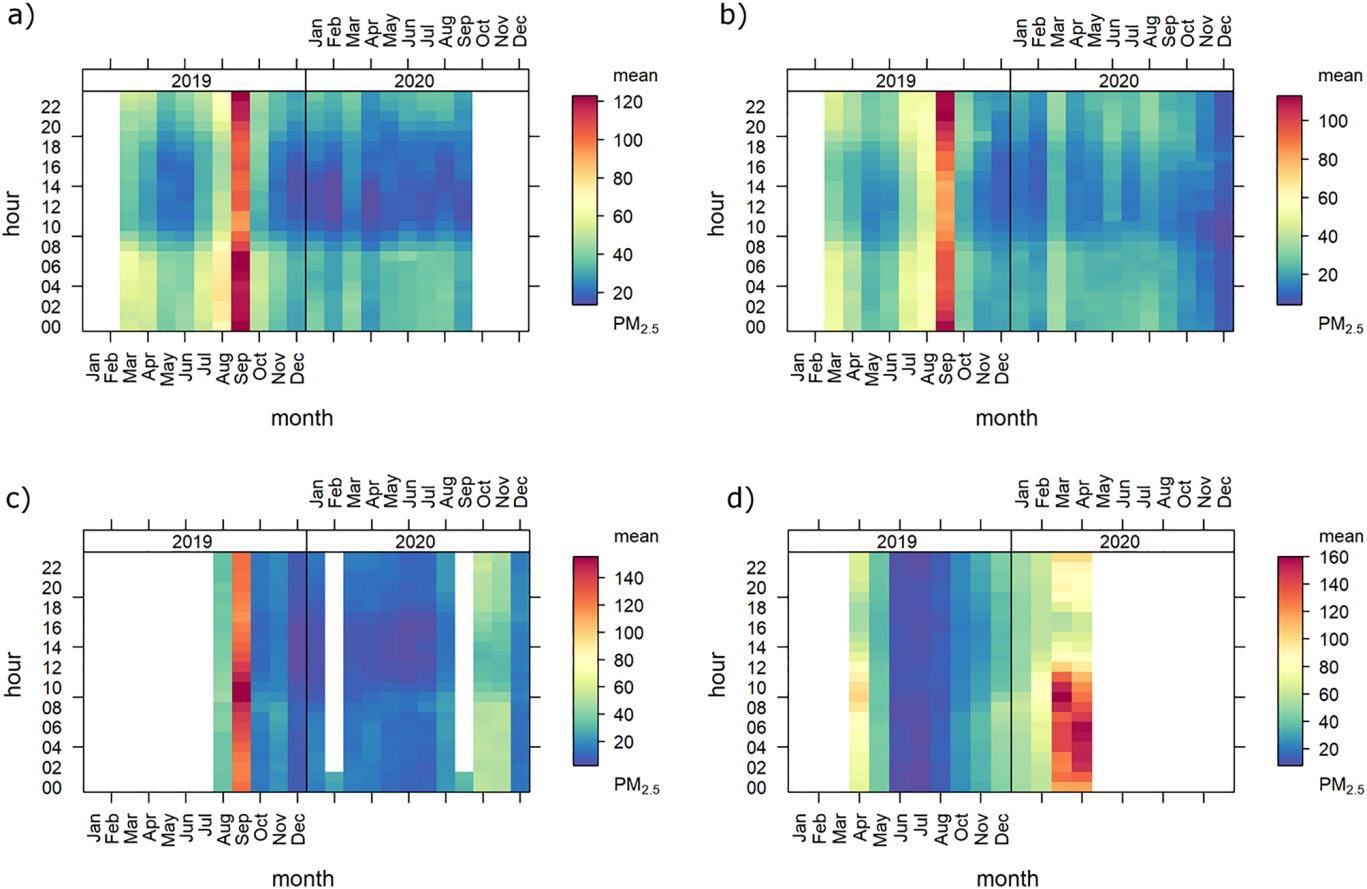
Monthly mean of PM_2.5_ concentration at (**a**) Putrajaya, (**b**) Bukit Fraser, (**c**) Kota Samarahan and (**d**) Chiang Mai.

Detailed monthly concentrations with hourly averages for PM_2.5_ are shown in Fig. 2, and for PM_10_ and PM_1_ in Figs. S1 and S2, respectively. It can be seen that in September 2019, the concentrations of PM_2.5_ started to increase from 10.00 pm until early morning (7.00 am) for Putrajaya, and the same is true for Bukit Fraser which recorded the highest average concentrations starting from 10.00 pm until midnight. Since Bukit Fraser is located near a mountain range, it is susceptible to being influenced by cold air masses during the night. According to Li et al.^29^, mountains area are more influenced by nighttime drainage flow, which resulted in the accumulation of cold air masses in the surface layer, which facilitated accumulation at night. For Kota Samarahan, the highest concentrations were determined in the morning around 8.00 am–10.00 am in September 2019. Chiang Mai had the highest PM_2.5_ concentrations in March and April 2020 with the highest concentrations around 9.00 am in March and around 6.00 am (early morning) in April. A stable boundary layer depth and mixing layer height combined with low wind speeds results in high PM concentrations in the morning in Chiang Mai^30,31^. The stability of mixing layer height, thereby resulting in the suppression of mixing layer evolution, and further increases PM concentrations near the ground surface in the morning^31^. The diurnal monthly variation of PM_2.5_ and PM_10_ reached up to 160 µg m^−3^ while for PM_1_ the diurnal monthly variations reached up to 100 µg m^−3^. Furthermore, the concentrations in June, July and August had the lowest PM_2.5_ values indicating the best air quality during these months for Chiang Mai.

### Haze days and normal days comparison

The concentrations of PM_10_, PM_2.5_ and PM_1_ are also compared for high pollution events (haze days) and normal days (Table S1), where if the 24 h daily average concentration of PM_2.5_ is > 75 µg m^−3^ the day is categorised as a haze day, as suggested by Zhang et al.^32^, while non-haze (normal days) have 24 h averages of PM_2.5_ < 50 µg m^−3^, as reported by Li et al.^33^. The average PM_10_, PM_2.5_ and PM_1_ concentrations during haze days were highest at Kota Samarahan with concentrations of 196 ± 59.5 µg m^−3^, 185 ± 57.2 µg m^−3^, and 108 ± 24.1 µg m^−3^ respectively, while for normal days, Putrajaya recorded the highest average concentrations of 33.2 ± 24.7 µg m^−3^, 29.5 ± 22.3 µg m^−3^ and 21.7 ± 14.7 µg m^−3^ for PM_10_, PM_2.5_ and PM_1_ respectively. Statistically, PM_10_, PM_2.5_ and PM_1_ varied significantly during haze days compared to normal days (*p* < 0.05, t-test). On average, the increments of PM_10_, PM_2.5_ and PM_1_ from normal days to haze days were 3.9 fold, 4.7 fold, 3.6 fold (Putrajaya); 5.4 fold, 5.7 fold and 4.8 fold (Bukit Fraser); 11.3 fold, 11.8 fold, 8.9 fold (Kota Samarahan); and 6.0 fold, 6.5 fold and 6.4 fold (Chiang Mai) respectively. During the haze days, all the measurement sites had average PM_2.5_/PM_10_ ratio values > 0.94 except Bukit Fraser. As Bukit Fraser is located further from the Malacca Straits, in the centre of Peninsular Malaysia and at a higher altitude, this is a possible explanation. Bukit Fraser had an average ratio of PM_2.5_/PM_10_ value of 0.87 ± 0.15 with a range of 0.82–0.91. On the contrary, lower ratio values of PM_1_/PM_2.5_ were observed during the haze days compared to normal days, where the average ratio of PM_1_/PM_2.5_ during haze days was 0.65 ± 0.03 for Putrajaya, 0.56 ± 0.10 for Bukit Fraser, 0.57 ± 0.04 for Kota Samarahan and 0.69 ± 0.04 for Chiang Mai compared to an average of 0.72 ± 0.11 for all measurement sites for normal days.

### Seasonal variation effects

The average concentrations of PM_10_, PM_2.5_ and PM_1_ based on different monsoons and seasons are listed in Table 1. The effect of monsoons and seasons at the monitoring sites is indicated by the fact that the average concentrations of PM_10_, PM_2.5_ and PM_1_ were the highest during the Southwest monsoon for all site in Malaysia and the dry season for Chiang Mai, but only PM_1_ had the highest average concentrations in Inter monsoon 2 for Bukit Fraser. The average concentrations of PM_10_, PM_2.5_ and PM_1_ during the dry season at Chiang Mai were 115 ± 75.5 µg m^−3^, 108 ± 74.5 µg m^−3^ and 77.0 ± 47.5 µg m^−3^, respectively. Lower concentrations of PM_10_, PM_2.5_ and PM_1_ were observed during the wet season in Chiang Mai compared to other seasons, while both Putrajaya and Bukit Fraser recorded the lowest concentrations during the Northeast monsoon, which is known as the high rainfall season. For Kota Samarahan, the Northeast monsoon and the Inter monsoon 1 values were similar, where the average concentration differences between the two monsoons were only 0.8 µg m^−3^ for PM_10_, 0.4 µg m^−3^ for PM_2.5_ and 0.2 µg m^−3^ for PM_1_.

**Table 1:** Average concentration of PM_10_, PM_2.5_ and PM_1_ based on different seasons (concentration in µg m^−3^).

	Inter monsoon 1 (March to May)	Southwest monsoon (end May to September)	Inter monsoon 2 (October to in the middle of November)	Northeast monsoon (November to March)
**Putrajaya**				
PM_10_	30.2 ± 18.8	63.8 ± 45.2	45.7 ± 22.4	29.9 ± 19.8
PM_2.5_	27.4 ± 15.9	58.1 ± 44.2	40.3 ± 20.1	26.8 ± 16.8
PM_1_	20.5 ± 10.1	39.7 ± 26.4	28.3 ± 12.8	19.6 ± 11.2
**Bukit Fraser**				
PM_10_	35.1 ± 21.9	61.0 ± 32.7	37.1 ± 32.5	24.1 ± 20.0
PM_2.5_	28.9 ± 12.9	52.9 ± 30.5	27.5 ± 23.9	18.7 ± 16.8
PM_1_	19.3 ± 7.40	31.6 ± 19.0	34.3 ± 13.4	11.5 ± 11.0
**Kota Samarahan**				
PM_10_	11.7 ± 9.23	111 ± 92.4	19.5 ± 14.7	12.5 ± 12.0
PM_2.5_	11.1 ± 8.63	104 ± 89.2	18.0 ± 12.6	11.5 ± 11.2
PM_1_	8.67 ± 8.00	63.4 ± 48.5	13.1 ± 8.46	8.47 ± 7.10

	Transition 1 (October to November)	Dry (December to March)	Transition 2 (April to May)	Wet (June to September)
**Chiang Mai**				
PM_10_	30.5 ± 14.1	115 ± 75.5	46.4 ± 18.1	12.0 ± 9.25
PM_2.5_	27.1 ± 10.9	108 ± 74.5	39.4 ± 15.3	10.9 ± 8.54
PM_1_	19.4 ± 7.14	77.0 ± 47.5	28.9 ± 11.9	8.08 ± 7.12

**Figure 3:**
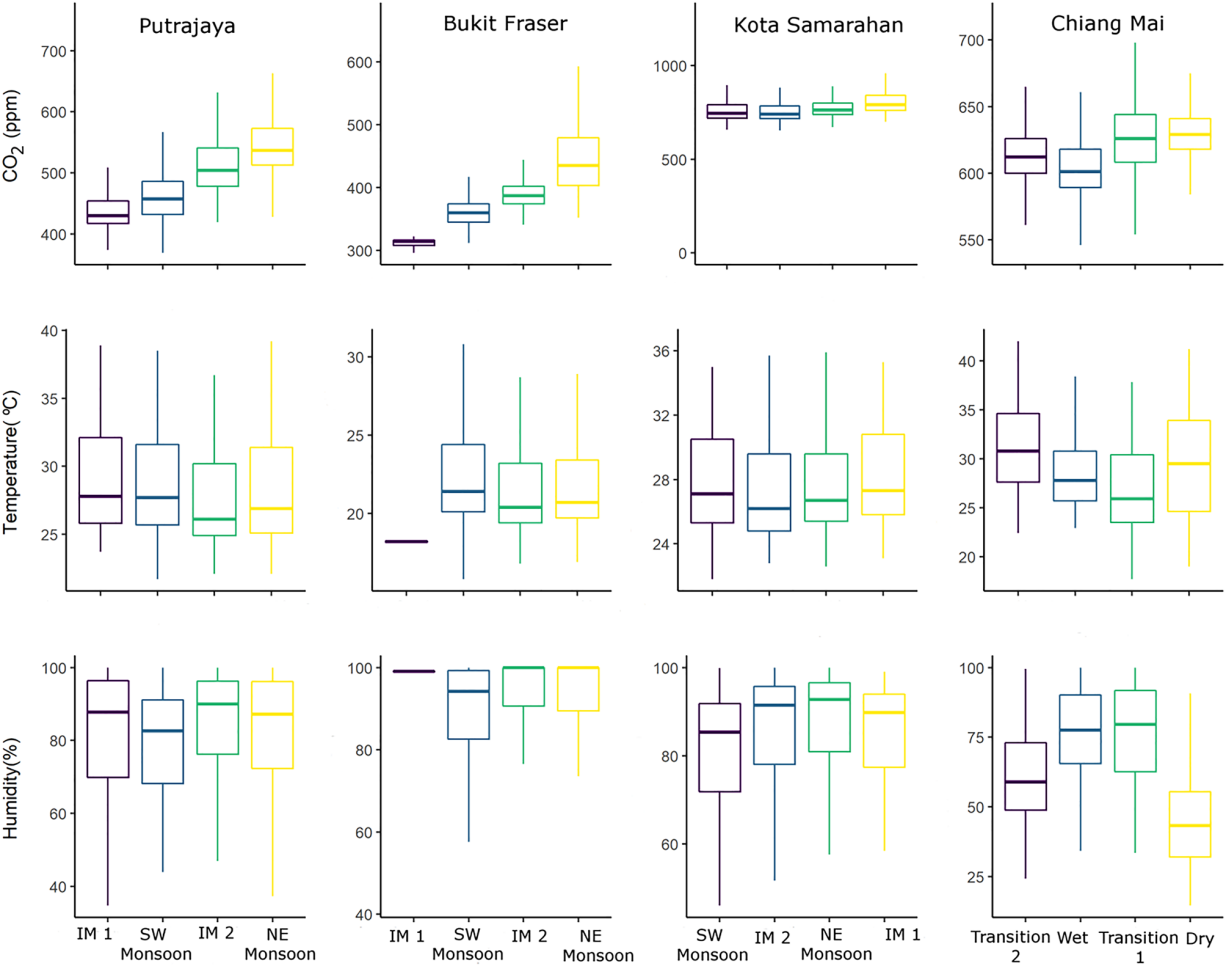
Seasonal variation of CO_2_, temperature and relative humidity based on different seasons in Putrajaya, Bukit Fraser, Kota Samarahan and Chiang Mai (IM 1, SW Monsoon, IM 2, and NE Monsoon represent Inter Monsoon 1, Southwest Monsoon, Inter Monsoon 2, and Northeast Monsoon respectively).

The effects of seasons and monsoons were also explored for CO_2_, temperature and relative humidity as shown in Fig. 3. For CO_2_, the highest average concentration was observed for Kota Samarahan during Inter monsoon 1 at 798 ± 51.7 ppm. For temperature, Putrajaya, Bukit Fraser and Kota Samarahan had average values below 30 °C during all monsoons, while for Chiang Mai, the highest average temperature was recorded during the Transition 2 season with the range 22.4–42.0 °C (average 30.9 ± 4.34 °C). The relative humidity pattern in Putrajaya was highest during Inter monsoon 2 (85.7 ± 12.4%), reaching almost 100% for all monsoons except the Southwest monsoon in Bukit Fraser, and the Northeast monsoon (88.1 ± 10.7%) in Kota Samarahan. Relative humidity in Chiang Mai showed variations in values where the Transition 1 season had the highest average value (76.5 ± 16.4%) and the lowest was recorded during the dry season (43.6 ± 14.2%).

To examine the relationships between PM_10_, PM_2.5_ and PM_1_ and the other parameters (temperature, relative humidity and CO_2_), a Pearson correlation matrix based on the different seasons was produced for monitoring sites (Figs. S3–S6). High positive correlations of all sizes of PM were observed for Chiang Mai in Transition 1, dry, Transition 2 and wet seasons (*r* > 0.96) while negative correlations were observed between relative humidity and temperature (*r* = − 0.89 for Transition 1 and dry season, *r* = − 0.94 for Transition 2, *r* = − 0.95 for wet season). For the other monitoring sites, significant relationships were observed between the PM parameters (PM_10_, PM_2.5_ and PM_1_) in Putrajaya and Kota Samarahan (*r* > 0.90) for all seasons. Bukit Fraser had lower correlations of PM parameters than Putrajaya and Kota Samarahan, but still had *r* > 0.74. Overall, no clear relationships were observed for temperature and relative humidity with PM_10_, PM_2.5_ and PM_1_ in all seasons.

### Air mass trajectory clustering

**Figure 4:**
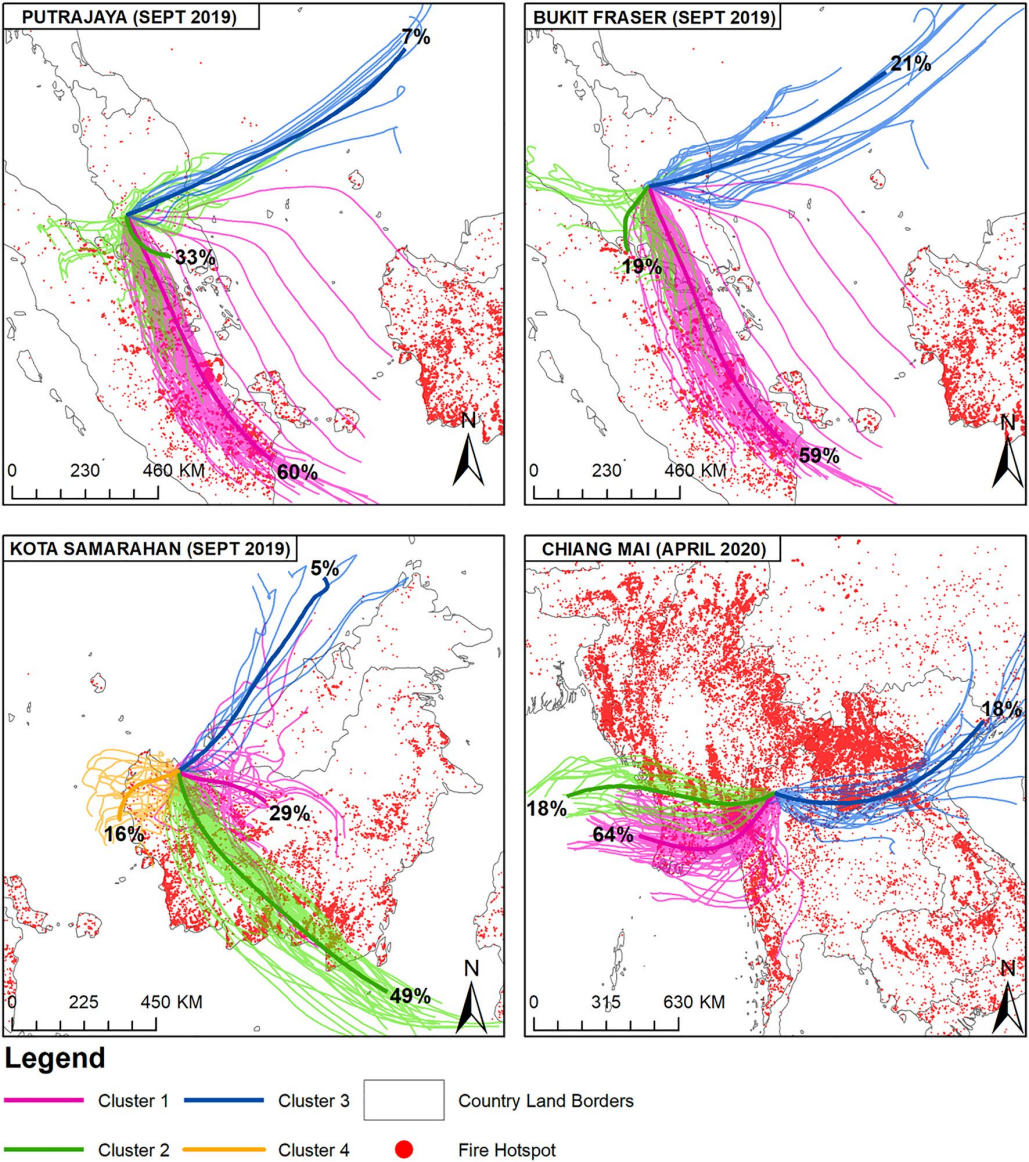
Cluster trajectory and hotspots distribution during haze days in Putrajaya, Bukit Fraser, Kota Samarahan and Chiang Mai. The maps were made using ArcMap v10.8.1 geospatial processing program http://www.esri.com and MODIS fire hotspot was downloaded from Aqua and Terra fire hotspot (https://firms.modaps.eosdis.nasa.gov/map/#d:24hrs;@0.0,0.0,3z). The map was produced by the author.

Figure 4 shows the backward trajectories and cluster analysis of the air masses for Putrajaya, Bukit Fraser and Kota Samarahan in September 2019 and for Chiang Mai in April 2020. All of the trajectories were analysed for the air masses with the highest concentrations of PM_10_, PM_2.5_ and PM_1_ during the monitoring duration, which happened during the Southwest monsoon. Clustering backward trajectory analysis shows that strong winds blew from the south of the Asian region in September 2019 (Southwest monsoon), bringing together smoke pollutants from the coastal area of Sumatra—about 60% of the air mass to Putrajaya and 59% of the air mass to Bukit Fraser. For Kota Samarahan, the dominant air mass (49%) was from the southeast, i.e. from southern Kalimantan where high numbers of hotspots were observed in the south coastal area of Kalimantan. Another 29% of the clustered air mass was identified as being from the east of Kota Samarahan and central Borneo Island. Cluster trajectory air masses determined for Chiang Mai were mainly associated with westerly winds from the coastal area that blew across the high number of biomass burning areas in Southern Myanmar. Other source contributions of air mass were from east of Chiang Mai that blew from southern Laos, also with a high number of hotspots. Another trajectory was mapped to see the contribution of air masses at the end of March 2020 (Fig. S7) where 62% of the air mass originated from the northwest of Chiang Mai, specifically from southern Myanmar.

### Deposition of particle in respiratory tract

Table 2 lists the deposition fraction visualisation in the human lung and deposition fraction values for the head, tracheobronchial (TB), and pulmonary regions. On average, the haze scenario had a higher maximum deposition value for all particle sizes than the non-haze scenario, where clear deposition of particle visualisation could be detected for PM_2.5_ when comparing both haze and non-haze scenarios. The individual total deposition fraction values (sum of the head, TB, and pulmonary) was higher for the haze scenario than on non-haze scenario for PM_10_, PM_2.5_, and PM_1_, suggesting higher particle deposition in the human lung on haze days than on non-haze days. The deposition fraction value was higher for the head compared to other regions, with values of 0.9387 (haze) and 0.9171 (non-haze) for PM_10_, 0.6211 (haze) and 0.4609 (non-haze) for PM_2.5_, and 0.2092 (haze) and 0.1361 (non-haze) for PM_1_. However, higher deposition of particles was observed during non-haze episodes than haze episodes for the TB, with a 2.2 fold increment for PM_10,_ a 1.6 fold increment for PM_2.5_, and a 1.2 fold increment for PM_1_. A higher deposition fraction value was observed for the pulmonary region during non-haze episodes than haze episodes, with a 40% increase for PM_10_, a 61% increase for PM_2.5_ and a 53% increase for PM_1_. When comparing the TB and pulmonary regions, it was found that PM_2.5_ had the highest deposition in these regions, and for both haze and non-haze scenarios, PM_1_ had the second-highest deposition fraction, while PM_10_ had the least deposition fraction for both the TB and pulmonary regions.

**Table 2:** Illustrations of particle deposition in human lungs; and deposition fraction values in the head, tracheobronchial (TB) and pulmonary regions during haze and non-haze scenarios.

	Deposition Fraction	
	Haze	Non-haze
PM_10_		
PM_2.5_		
PM_1_		

## Discussion

When compared to the 2005 World Health Organization’s (WHO) air quality guidelines and the US Environmental Protection Agency (USEPA) National Air Quality Standards, the average concentrations of PM_2.5_ at all measurement sites for the whole measurement period exceeded the WHO’s guidelines of 25 µg m^−3^, while Putrajaya and Chiang Mai slightly exceeded the USEPA standard of 35 µg m^−3^. Kota Samarahan had the lowest PM_10_ and PM_2.5_ average concentrations while Bukit Fraser had the lowest PM_1_ average concentration for the whole measurement campaign. The variabilities of PM_10_ and PM_2.5_ concentrations between sites and locations was due to the source contributions, with PM_10_ being suggested to be likely from local origins^34^ while PM_2.5_ being affected by multiple factor such as meteorological parameters, seasonal effects, and biomass burning source^8^. Interesting pattern of high pollution PM concentrations in Chiang Mai was suggested to be during the worldwide COVID-19 pandemic and lockdown, which did not play a significant part in PM reduction. Moreover, another peak concentration was observed in early April 2019 for Chiang Mai which is consistent with the finding by Yabueng et al.^3^, who reported a peak in PM_2.5_ concentration during the middle of March to April 2019. It also can be seen that the monthly concentrations of PM_10_, PM_2.5_ and PM_1_ in Malaysia do not vary very much throughout the measurement duration which can be suggested to be due to COVID-19 lockdown impacts. As reported by Othman and Latif^35^, there was a significant reduction of PM_10_ and PM_2.5_ after the implementation of the control movement order (MCO) (lockdown) in Malaysia where reduced concentrations of PM were observed during the MCO that started in the middle of March 2020.

The increment of PM_2.5_ from a normal day to haze day is the highest compared to PM_10_ and PM_1_. This can be due to PM_2.5_ being the most dominant pollutant produced during biomass burning, also indicated by the high ratio value of PM_2.5_ to PM_10_ (PM_2.5_/PM_10_). The higher ratio values for other monitoring site indicate the influence of the transboundary effect and biomass burning during haze days and additional local contributions, which is consistent with previous studies^36–38^. On normal days, the ratio values of PM_2.5_/PM_10_ in central Peninsular Malaysia were recorded as average values of 0.81 ± 0.80 by Othman et al.^6^ which is similar to the result of this study, while studies by Rupakheti et al.^39^ and Yin et al.^40^ had lower PM_2.5_/PM_10_ in northwestern and southwestern China which is influenced by the coarse PM fraction from sand and dust storms. Lower PM_1_/PM_2.5_ during haze days compared to normal days indicates lower concentrations of PM_1_ compared to PM_2.5_ during haze days and that PM_1_ is not dominantly generated from biomass burning. Thus, other sources of PM_1_ can be suggested to be industrial emissions, motor vehicle emissions and coal combustion which are also linked to rapid economic development^41,42^. Moreover, as reported by Lee et al.^43^, the best indicator for vehicular emissions can be found to be PM_1_ for areas with high emissions from motor vehicle compared to PM_2.5_.

Higher concentrations of all sizes of PM during the dry season for Chiang Mai compared to other monitoring sites could be due to an increase in particle pollution during this season. A study by Pengchai et al.^44^ found high concentrations of PM_10_ in the dry season in the Northern part of Thailand. Moreover, PM concentrations were suggested to be high during the dry season and then decreased in the wet season which is related to the wash-out effect, where highest precipitation was observed in August–September^4^. In the case of the Southwest monsoon in Malaysia, normally there are still some rainfall events, especially in the beginning (May and June) of the monsoon, which may affect the PM concentrations. The rainfall pattern in the central region of Peninsular Malaysia was 765 mm year^−1^ with 35% of the rainfall during the whole the Southwest monsoon, a decreasing trend of monthly rainfall throughout the monsoon^45^ and a deficit of rainfall allowing the accumulation of hotspots starting from June^46^. The lowest temperature value during the dry season was also observed by Pongpiachan and Paowa^47^ which shows the dry season does not particularly relate to high temperature. Moreover, a stronger correlation between all sizes of PM for Putrajaya and Kota Samarahan compared to Bukit Fraser indicated the effects of urban anthropogenic sources. The relationship between PM_10_ and PM_2.5_ was stronger in the urban area, suggesting that both particle sizes are influenced by anthropogenic activities, but different sites and locations also play significant roles in the correlation between PM_10_ and PM_2.5_^48^.

A high number of hotspots in coastal areas of Sumatra were suggested to be peatland fires that are usually associated with low rainfall during the Southwest monsoon and which also impact other areas such as Singapore and Southern Thailand. This result is consistent with previous studies^6,7,46^. Haze episodes in Southeast Asia, especially Malaysia, are governed by a general wind direction and patterns from the south^49^. It can be said that high numbers of hotspots around Chiang Mai including in Myanmar and Laos contribute to high PM concentrations in this centre of Southeast Asia. As suggested by Pimonsree and Vongruang^38^, major emissions of PM were spotted in March when the contribution of biomass burning was found to be approximately 85% and 89% for PM_10_ and PM_2.5_ respectively in the centre of Southeast Asia region with strong PM concentration gradients from the biomass burning source within 50 km.

In terms of health effects, it can be suggested that all sizes of PM were highly deposited in the head when inhaling of air. This is due to the facts that 90% of air is inhaled by humans through their noses, which are located in the head^50^. There was also a higher deposition of particles during haze compared to non-haze days especially for coarse particle while fine particles (PM_2.5_ and PM_1_), higher deposition occurred during non-haze but still total deposition fraction value were higher during haze episode. Long et al.^51^ observed a significantly higher deposition fraction in the head compared to TB and pulmonary for particle sizes ranging from 0.43 µm to much larger sizes, as well as a significantly lower deposition fraction during haze days compared to non-haze days. It has been suggested that smaller particles enter and accumulate in the innermost reaches of the human respiratory system, where prolonged exposure to small particles such as ultrafine and nanoparticles may have adverse human effects^52^. Additionally, carcinogenic metals bound to PM_2.5_ such as arsenic (As), cadmium (Cd), Cobalt (Co), chromium (Cr) and nickel (Ni) were found to be higher in human lung fluid during haze days compared to non-haze days, with Cr having the highest cancer risk value, followed by As^53^. Thus, it is clearly shown that haze episodes have a detrimental effect on human health. It is recommended that outdoor activities be limited during high pollution days.

## Conclusion

This study used monitoring data of PM_10_, PM_2.5_ and PM_1_ concentrations from four monitoring sites in Malaysia and Thailand revealing that all sizes of PM had similar trends in concentrations during the monitoring duration. The highest daily mean concentrations were observed for Kota Samarahan (Malaysia) in September, which was identified as a haze event, with average daily average concentrations of 196 ± 59.5 µg m^−3^ for PM_10_, 185 ± 57.2 µg m^−3^ for PM_2.5_ and 108 ± 24.1 µg m^−3^ for PM_1_. For Chiang Mai (Thailand), the high concentration of all size of PM was recorded in end of March and peak concentration in early of April 2020. An increment of PM_2.5_ concentration was observed during haze days where the PM_2.5_/PM_10_ ratio value was close to 1, indicating that PM_2.5_ was significantly contributed to the haze episode. Air mass trajectories coupled with hotspots data clearly show that contributions of all sizes of PM in Putrajaya, Bukit Fraser and Kota Samarahan were from biomass burning, particularly in Sumatra and Kalimantan. The westerly air masses which coincided with high numbers of hotspots related to biomass burning activity in the northern Southeast Asia region was suggested to be the source of all sizes of PM in Chiang Mai during the dry season. Total deposition of particles in the human respiratory tract for outdoor exposure was observed to be higher during haze compared to non-haze, indicating that human health is severely impacted during haze episodes. Further studies investigating the human health impacts of high concentrations of PM need to be undertaken to look at the combined overall impact of PM in the Southeast Asia region.

## Methods

### Study location

Putrajaya, Bukit Fraser and Kota Samarahan in Malaysia and Chiang Mai in Thailand were the monitoring sites used for this study. Both Chiang Mai and Kota Samarahan are usually associated with high concentrations of PM and haze episodes during the dry season. Chiang Mai, located in northern Thailand, is a basin surrounded by mountain ranges with 30% agricultural areas and is also close to biomass burning sources^3^. Putrajaya and Bukit Fraser are chosen as the monitoring sites due to the variation of PM concentrations at these sites, where Putrajaya is impacted by anthropogenic activities from the Kuala Lumpur urban environment and also transport of pollutants from Sumatra, Indonesia. Bukit Fraser, which located in the mountainous terrain of Titiwangsa, is in the centre of Peninsular Malaysia. Measurements of PM in Bukit Fraser will provide insights into the impact of seasons and the long-range transport of pollutants to this high altitude site. Thus spatial–temporal analysis of the different sizes of PM could help in determining the association of PM pollution with different seasons in the Southeast Asia region. Additional information and characteristics of each location are provided in Table S2 and Fig. S8.

### PM data and monitoring

The concentration of PM_10_, PM_2.5_ and PM_1_ with other parameters such as CO_2_, temperature and relative humidity were monitored over one year period in 2019 and 2020 using the AS-LUNG V.2 outdoor sensing device. The measurement campaigns were different for each site where the duration of the measurements was typically for more than one year, aiming to include all seasons and monsoons. A measurement of CO_2_ was performed as an indicator for air pollutant level in the study location, where high level of CO_2_ could suggest high air level of air pollution in the surrounding of the study location. The AS-LUNG V.2 was built in with sensors for PM (PMS3003, Plantower, Beijing, China), CO_2_ (S8, Senseair AB, Sweden), and temperature/relative humidity (SHT31, SENSIRION, Staefa ZH, Switzerland) that were installed inside a waterproof housing that was powered by a solar panel. As for backup, eight batteries with 10,000 mAH capacity were also connected to the power outlet to provide sufficient power for the sensor in the event that the solar panel received insufficient power. The weight of the sensor was about 5 kg with the waterproof housing weighing about 1.2 kg, and measuring 60 cm × 50 cm × 50 cm. The sensors were programmed to measure all parameters for every 15 s, and the data was saved to an SD card. AS-LUNG V.2 sensor is small device with no noise; compact outer case and easily set-up for outdoor measurement; and evaluated against research-grade instruments with coefficient of determination (*R*^2^) almost 0.895–0.998 that indicated this sensor are qualified for research^54^.

At each monitoring site, the AS-LUNG V.2 sensors were installed at high level locations. In Chiang Mai, the sensor was installed on the rooftop of four-storey building, while in Putrajaya, the sensor was installed on top of an air quality cabin about 5 m above the ground. In Kota Samarahan, the sensor was installed on the second floor of a university building which far away from the parking lot and human interference while in Bukit Fraser, the sensor was installed on a light pole about 2 m from the ground on a site that is a campus site and not open to the public. The seasons in Chiang Mai are described as the wet season (June to September), Transition 1 (October to November), dry season (December to March) and Transition 2 (April to May)^44,55^. For seasons in Malaysia, Inter monsoon 1 (March to May), Southwest monsoon (end of May to September), Inter monsoon 2 (October to in the middle of November) and Northeast monsoon (November to March) were applied for this study.

Data quality assurance and quality control (QA/QC) were conducted with the data from the Continuous Air Quality Monitoring Station for PM_10_ and PM_2.5_ for hourly data which indicated that the sensor data is about ± 30% different and the *R*^2^ value > 0.6. Due to the lack of data for PM_1_ measured by the Continuous Air Quality Monitoring Station, the data accuracy for PM_1_ was derived from Lung et al.^56^ who conducted a side-by-side comparison between the AS-LUNG V.2 sensor and the GRIMM instrument to determine the *R*^2^. The *R*^2^ value for PM_1_ appeared to be high, ranging between 0.931 and 0.996. Statistical analysis for the obtained data is performed using R software with the Openair package.

### Backward air mass trajectory analysis

The Hybrid Single-Particle Lagrangian Integrated Trajectory (HYSPLIT) with analysis of cluster backward trajectories was used to investigate air mass trajectories for high concentrations of PM at each of the measurement sites from 2019 to 2020. The metrological data input was obtained from the Global Data Assimilation System (GDAS) with a height of 500 m, which was then assessed for the daily run. Following the completion of daily runs, standard clustering was analysed for 36 h to cluster with an input of four number of clusters. The airmass trajectory was then overlaid with Moderate Resolution Imaging Spectroradiometer (MODIS) satellite data on the Aqua and Terra fire hotspot (https://firms.modaps.eosdis.nasa.gov/map/#d:24hrs;@0.0,0.0,3z) with confidence values ranging from 0 to 100%.

### Human airway particle dosimetry

The deposition fraction of all particle sizes in the human respiratory tract was modelled using Multiple-path Particle Dosimetry software (MPPD, v3.04), which was used to gain a clear understanding on particle deposition in the human respiratory system. Basically, this MPPD was based on single-path and multiple-path methods for tracking air flow and calculating aerosol deposition in the human lung where the single-path method calculates deposition along a typical path per airway generation, whereas the multiple-path method calculates particle deposition along all airways of the lung and provides lobar-specific and airway-specific information^57^. This software was developed by Applied Research Associates, Inc., where it is usually applied to calculate the deposition and clearance of monodisperse and polydisperse aerosols in the respiratory tract. In this study, the input data used to run the software was restricted to human morphology using the Yeh/Schum Symmetric Model, with functional residual capacity (FRC) and Upper Respiratory Tract (URT) volume set to default values of 3300 mL and 50 mL, respectively. For particle properties’ input data, the sizes of PM_10,_ PM_2.5_, and PM_1_ were inputted as 10, 25, and 1, respectively, for the count median diameters (CMD). The steps and input data selection for this software were performed as following Manojkumar et al.^58^. In this study, the PM_10_, PM_2.5_, and PM_1_ average concentrations across all sites were used as input data for aerosol concentrations to represent the outdoor exposure of the individual adult during both haze and non-haze (normal day) scenarios, without regard for geographical and meteorological conditions.

## Supplementary Information

Supplementary Information.
