# Supplementary material for: Spatial–temporal variability and health impact of particulate matter during a 2019–2020 biomass burning event in Southeast Asia
Source: Sci Rep. 2022 May 10;12:7630. doi: 10.1038/s41598-022-11409-z (PMC9086666; doi:10.1038/s41598-022-11409-z)
Supplement: Supplementary file 1 — Supplementary Information. [file 41598_2022_11409_MOESM1_ESM.docx]

Table S1 Average concentration of PM_10_, PM_2.5_ and PM_1_ (concentration in µg m^-3^) during haze, normal day and whole measurement

|  | Condition | PM_10_ | | | PM_2.5_ | | | PM_1_ | | | PM_2.5_/PM_10_ | | | PM_1_/PM_2.5_ | | |
| --- | --- | --- | --- | --- | --- | --- | --- | --- | --- | --- | --- | --- | --- | --- | --- | --- |
|  |  | Min | Max | Average | Min | Max | Average | Min | Max | Average | Min | Max | Average | Min | Max | Average |
| Putrajaya | Haze | 79.7 | 217 | 131±37.9 | 75.0 | 208 | 126±36.0 | 51.9 | 123 | 80.0±18.7 | 0.92 | 0.97 | 0.96±0.01 | 0.59 | 0.72 | 0.65±0.03 |
|  | Normal day | 7.08 | 58.8 | 33.2±24.7 | 6.88 | 49.9 | 29.5±22.3 | 5.74 | 35.8 | 21.7±14.7 | 0.82 | 0.99 | 0.89±0.20 | 0.64 | 0.84 | 0.74±0.08 |
|  | Whole measurement | 7.08 | 217 | 41.2±31.8 | 6.88 | 208 | 37.1±29.7 | 5.74 | 123 | 26.5±18.4 | 0.82 | 0.99 | 0.91±0.07 | 0.59 | 0.84 | 0.75±0.08 |
|  |  |  |  |  |  |  |  |  |  |  |  |  |  |  |  |  |
| Bukit Fraser | Haze | 88.6 | 238 | 144±46.7 | 79.2 | 203 | 127±40.5 | 45.2 | 102 | 70.5±19.0 | 0.82 | 0.91 | 0.88±0.18 | 0.50 | 0.61 | 0.56±0.10 |
|  | Normal day | 1.11 | 64.0 | 26.4±26.0 | 0.62 | 49.9 | 21.9±21.0 | 0.33 | 31.1 | 14.4±12.7 | 0.40 | 0.97 | 0.87±0.15 | 0.28 | 0.65 | 0.68±0.15 |
|  | Whole measurement | 1.11 | 238 | 35.8±34.5 | 0.62 | 203 | 33.0±30.3 | 0.33 | 102 | 19.4±19.0 | 0.40 | 0.97 | 0.88±0.13 | 0.28 | 0.65 | 0.69±0.13 |
|  |  |  |  |  |  |  |  |  |  |  |  |  |  |  |  |  |
| Kota Samarahan | Haze | 86.6 | 289 | 196±59.5 | 80.6 | 278 | 185±57.2 | 51.9 | 156 | 108±24.1 | 0.83 | 0.97 | 0.95±0.02 | 0.51 | 0.70 | 0.57±0.04 |
|  | Normal day | 1.19 | 61.4 | 17.3±13.6 | 0.99 | 49.8 | 15.6±11.6 | 0.73 | 46.1 | 12.1±9.50 | 0.72 | 0.97 | 0.91±0.12 | 0.59 | 0.93 | 0.76±0.15 |
|  | Whole measurement | 1.19 | 289 | 29.6±28.4 | 0.99 | 278 | 27.5±25.0 | 0.73 | 156 | 20.3±20.1 | 0.72 | 0.97 | 0.95±0.08 | 0.51 | 0.93 | 0.78±0.11 |
|  |  |  |  |  |  |  |  |  |  |  |  |  |  |  |  |  |
| Chiang Mai | Haze | 83.7 | 216 | 141±42.1 | 78.3 | 209 | 135±41.4 | 57.2 | 140 | 95.4±26.6 | 0.92 | 0.96 | 0.96±0.01 | 0.66 | 0.74 | 0.69±0.04 |
|  | Normal day | 3.24 | 61.1 | 23.3±31.5 | 2.74 | 49.9 | 20.5±29.5 | 1.72 | 37.7 | 14.9±21.6 | 0.80 | 0.95 | 0.89±0.08 | 0.61 | 0.79 | 0.72±0.09 |
|  | Whole measurement | 3.24 | 216 | 39.1±38.0 | 2.74 | 209 | 35.6±34.5 | 1.72 | 140 | 26.1±24.5 | 0.80 | 0.96 | 0.90±0.07 | 0.61 | 0.79 | 0.73±0.07 |


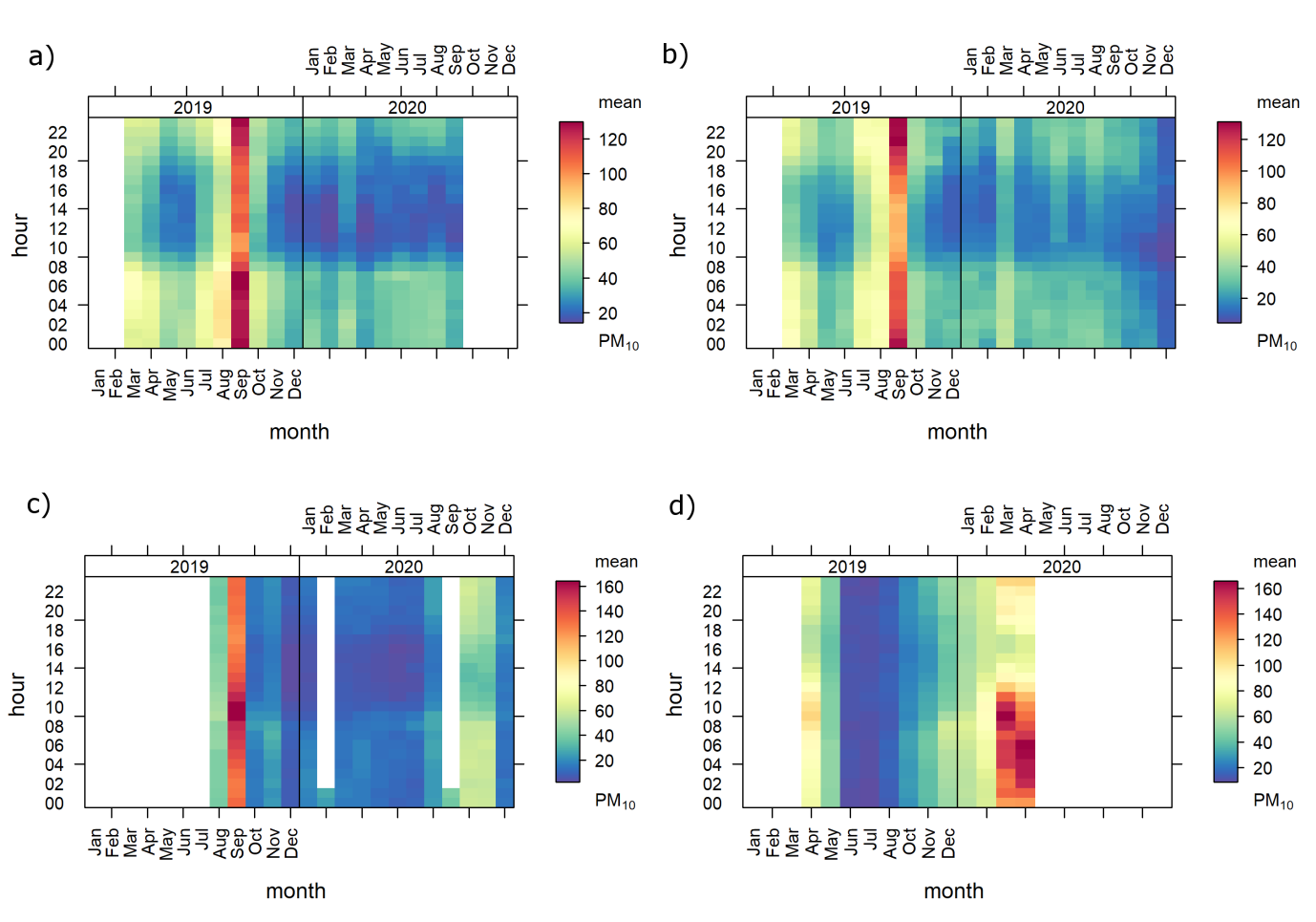


Figure S1 Monthly mean of PM_10_ concentration at a). Putrajaya, b). Bukit Fraser, c). Kota Samarahan and d). Chiang Mai


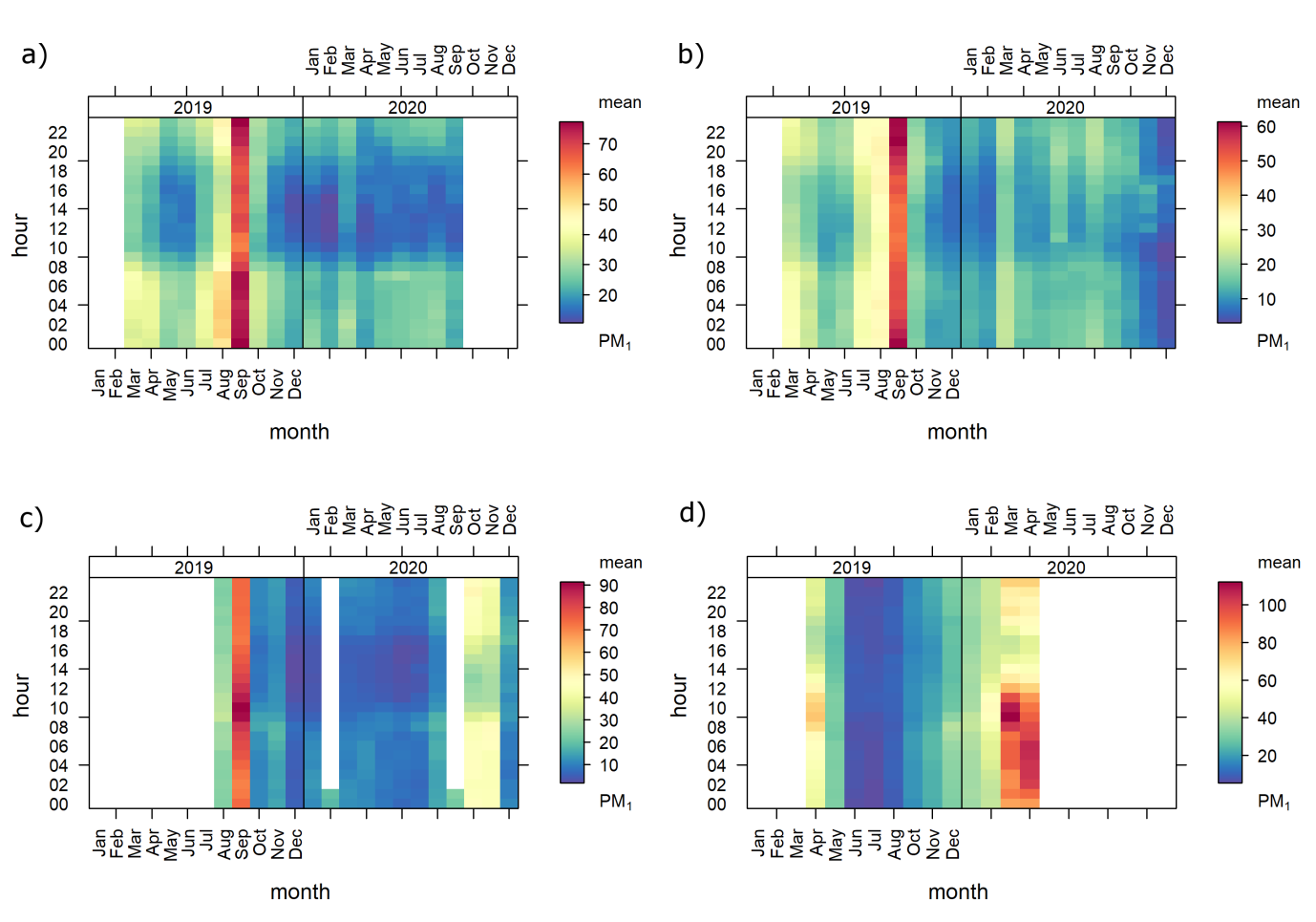


Figure S2 Monthly mean of PM_1_ concentration at a). Putrajaya, b). Bukit Fraser, c). Kota Samarahan and d). Chiang Mai


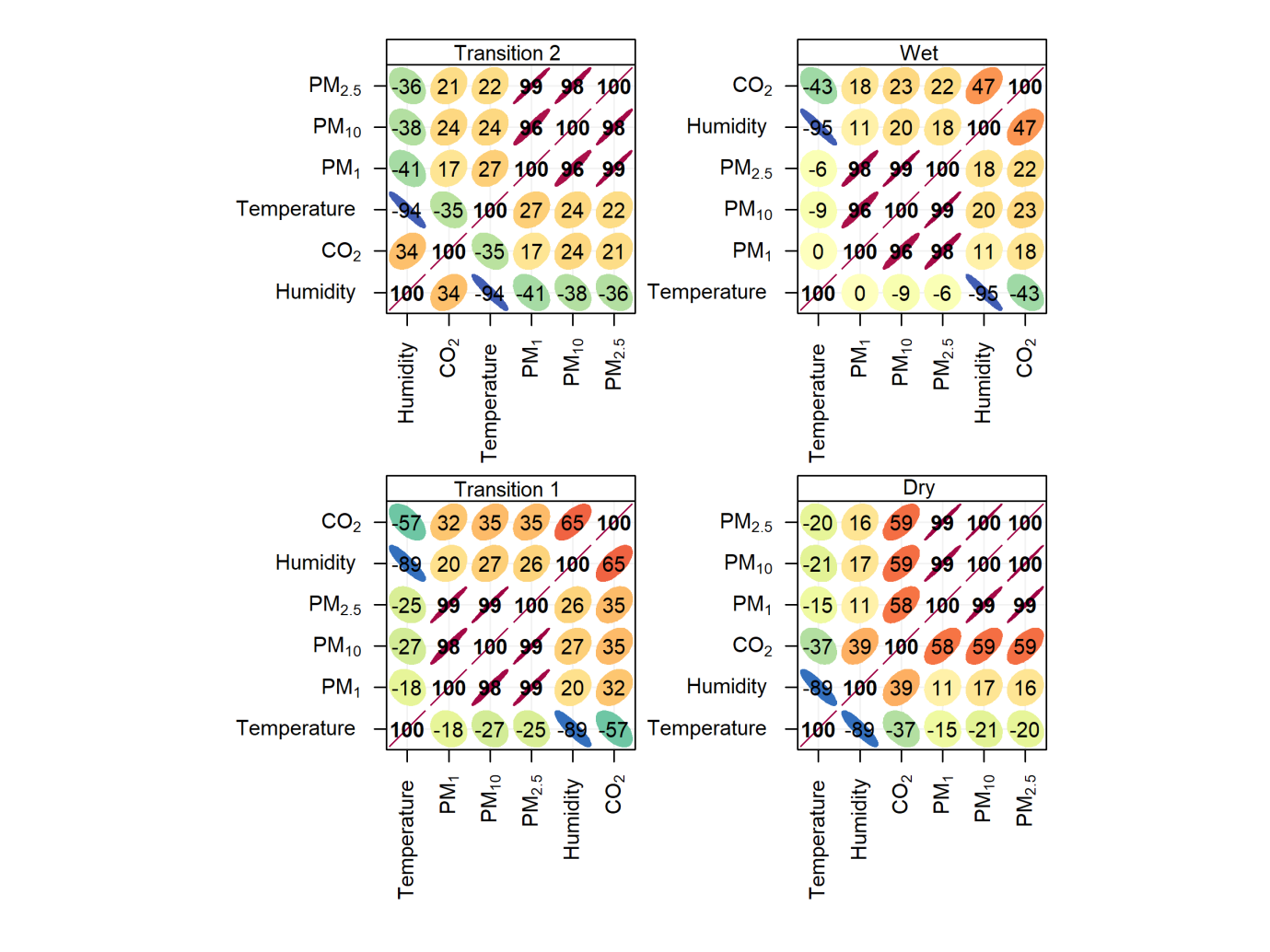


Figure S3 Correlation matrix of PM, temperature, relative humidity and CO_2_ based on different seasons in Chiang Mai


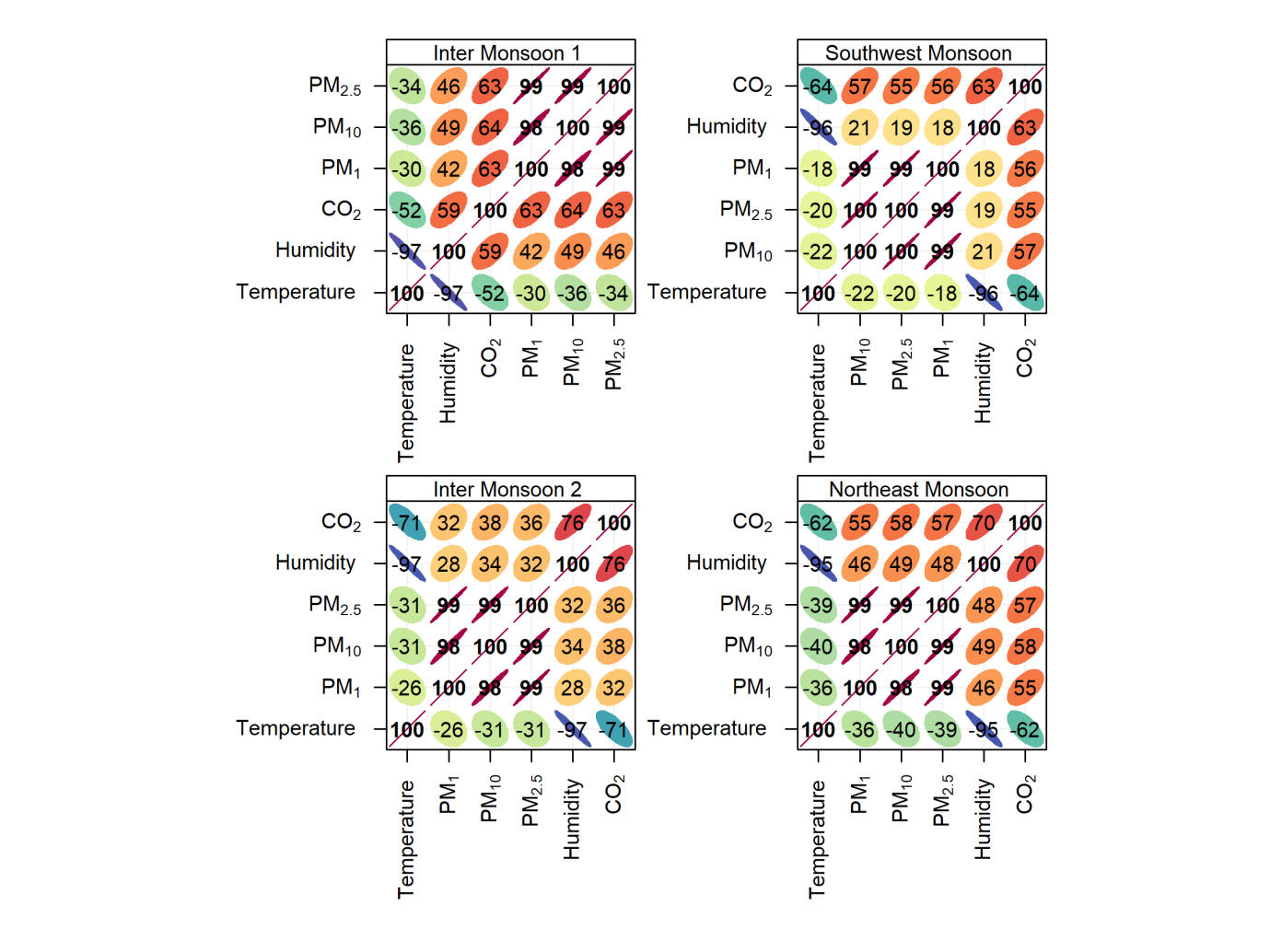


Figure S4 Correlation matrix of PM, temperature, relative humidity and CO_2_ based on different seasons in Putrajaya


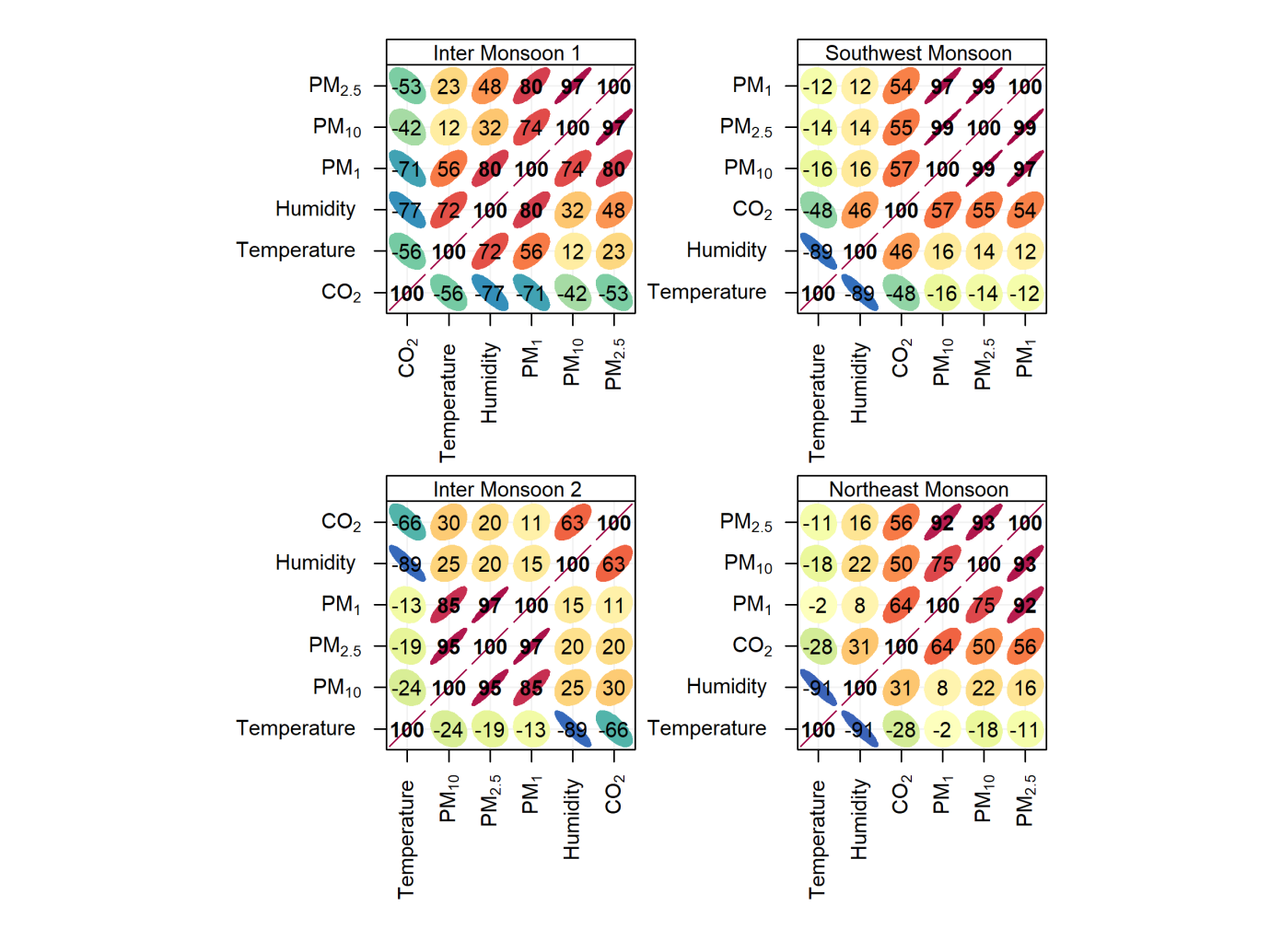


Figure S5 Correlation matrix of PM, temperature, relative humidity and CO_2_ based on different seasons in Bukit Fraser


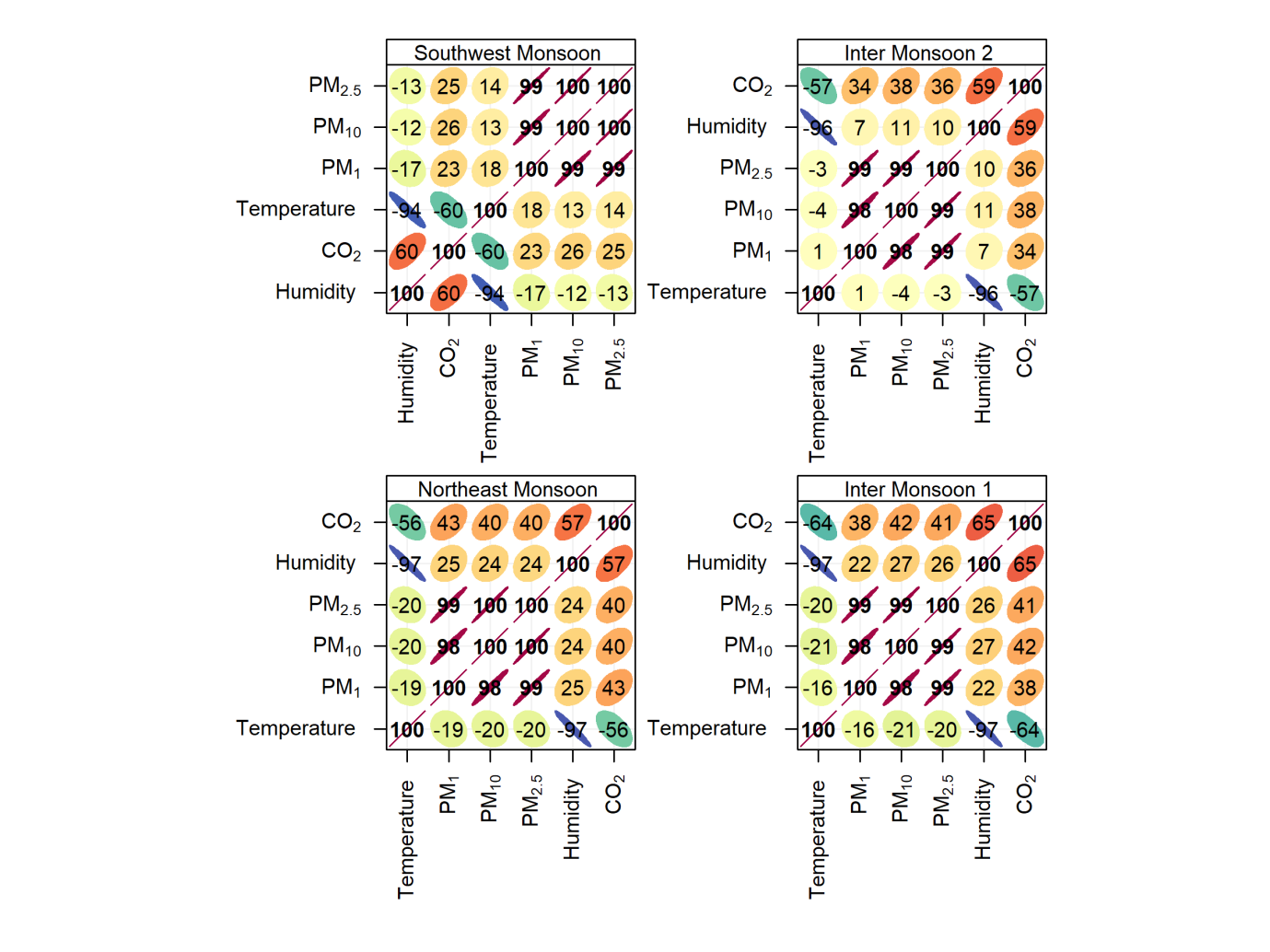


Figure S6 Correlation matrix of PM, temperature, relative humidity and CO_2_ based on different seasons in Kota Samarahan


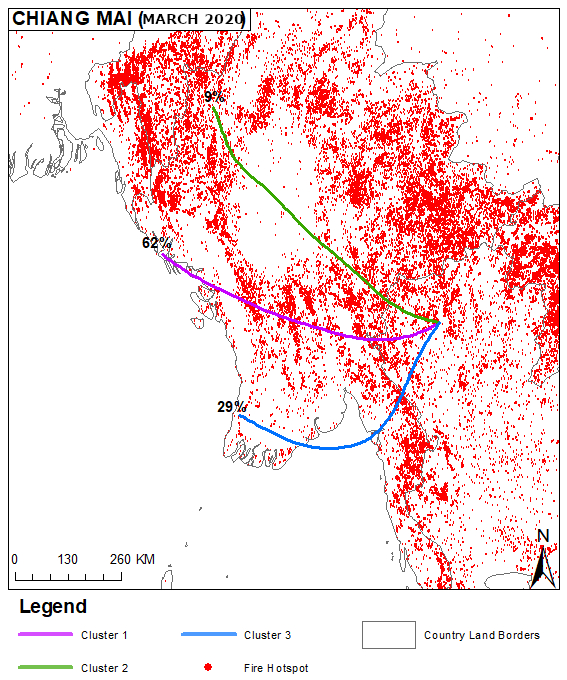


Figure S7 Backward cluster trajectory of air mass at Chiang Mai on March 2020. The map was made using ArcMap v10.8.1 geospatial processing program <http://www.esri.com> and MODIS fire hotspot was downloaded from Aqua and Terra fire hotspot (<https://firms.modaps.eosdis.nasa.gov/map/#d:24hrs;@0.0,0.0,3z>). The map was produced by the author.

Table S2 Location of PM measurement sites in Malaysia and Thailand

| Site | Longitude latitude | Location of the instrument | Sampling duration | Characteristics |
| --- | --- | --- | --- | --- |
| Putrajaya, Malaysia | 2°54'55.36"N, 101°41'25.81"E | On a rooftop of air measurement cabin own by Department of Environment. Near to school, residential and office building. | 23rd March 2019 until 24th September 2020 | Administrative city of Malaysia which is a focus point for administrative, conference and any national events. Located about 30 km to Kuala Lumpur International Airport and 36 km to Kuala Lumpur City Centre. |
| Bukit Fraser, Malaysia | 3°43'30.05"N, 101°42'59.37"E | On a post about 2 m from the ground. Remote area and rural area | 16th March 2019 until 31st December 2020 | Tourist attraction in mountain rural area. Surrounded by densely tropical forest. |
| Kota Samarahan, Malaysia | 1°26'49.52"N, 110°27'10.20"E | Second floor of university building. Near to residential area | 21st August 2019 until 23rd December 2020 | A city under Sarawak State in Borneo. Located near to Indonesian Borneo border that rich with huge land of peat soil. |
| Chiang Mai, Thailand | 18°47'42.71"N, 98°57'29.53"E | Roof top of four story university building. Near to the city and business area | 1st April 2019 until 24th September 2020 | A city in Northern of Thailand which near to Myanmar and Laos. |


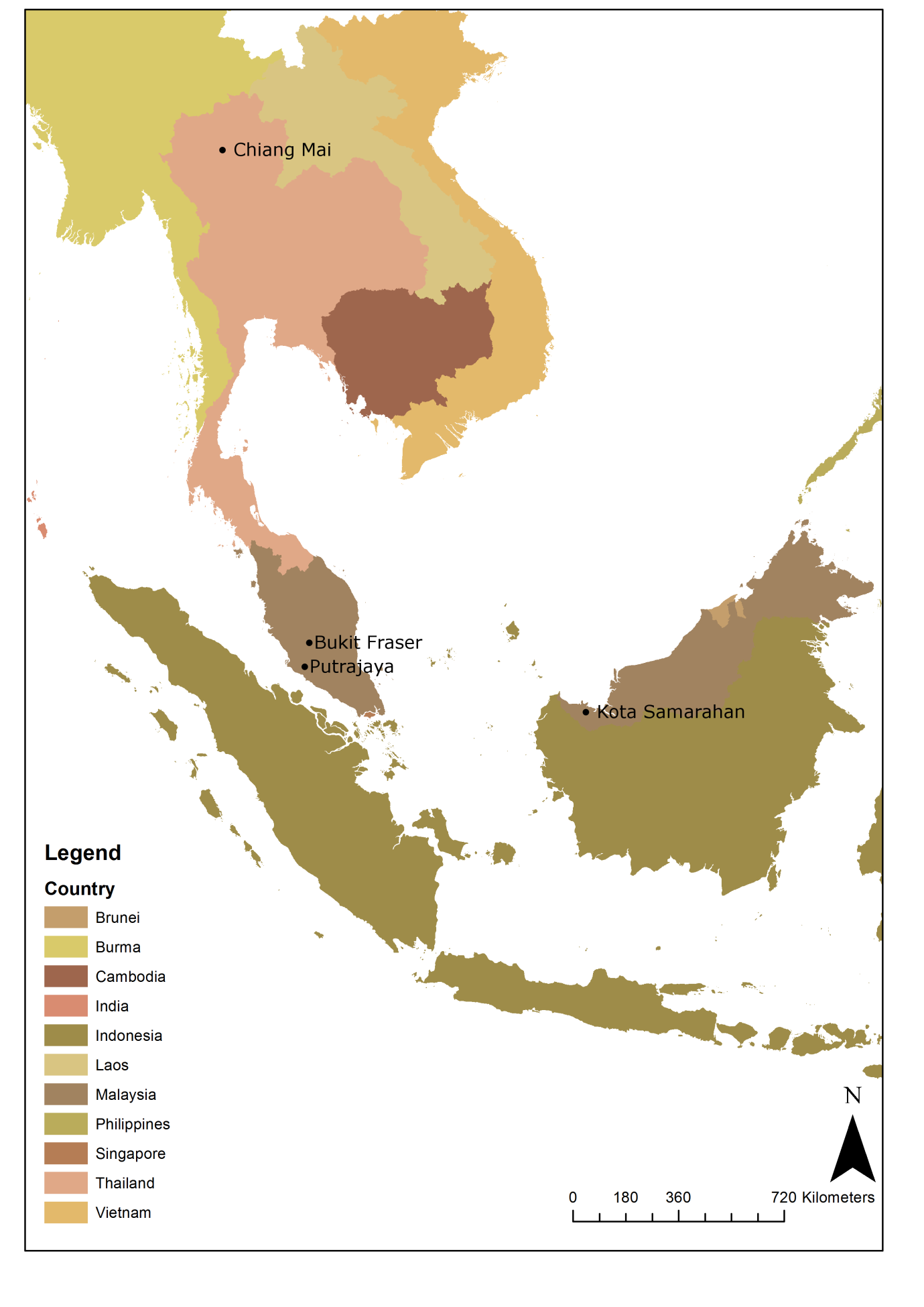
Figure S8 Location of measurement sites in Putrajaya, Bukit Fraser and Kota Samarahan (Malaysia) and Chiang Mai (Thailand). The map was made using ArcMap v10.8.1 geospatial processing program <http://www.esri.com> by the author.
